# Evaluation of the Nutritional Education Program in Increasing Nutrition-Related Knowledge in a Group of Girls Aged 10–12 Years from Ballet School and Artistic Gymnastics Classes

**DOI:** 10.3390/nu17091468

**Published:** 2025-04-26

**Authors:** Magdalena Leonkiewicz, Agata Wawrzyniak

**Affiliations:** Department of Human Nutrition, Institute of Human Nutrition Sciences, Warsaw University of Life Sciences (WULS-SGGW), 159C Nowoursynowska Str., 02-776 Warsaw, Poland; magdalena.leonkiewicz@gmail.com

**Keywords:** nutritional education program, ballet dancers, artistic gymnasts, age 10–12 years

## Abstract

**Background:** Adherence to nutritional recommendations in groups of adolescents practicing various sports, including esthetic disciplines, is insufficient. Hence, the authors of this study attempted to design, implement and evaluate a nutritional education program for girls aged 10–12 attending a ballet school and artistic gymnastics classes. **Methods**: The study was conducted with 60 female students at the state ballet school and artistic gymnastics classes (professionally practicing ballet and artistic gymnastics). The nutritional education program was implemented by all students for a period of 4 weeks. The program consisted of three parts: group sharing and discussing the educational brochure, group nutritional workshops, and providing and discussing individual nutritional recommendations. Information provided to students during the nutritional education program concerned the principles of proper nutrition contained in the Pyramid of Healthy Nutrition and Physical Activity for Children and Youth, the most important sources of nutrients in the diet and their role, and the principles of nutrition of people practicing sports/training. Before starting the nutritional education program and 3 months after its completion, the level of nutritional knowledge was assessed in the group of ballerinas and artistic gymnasts to evaluate the program. **Results**: The proposed nutritional education program had a significant impact on the level of nutritional knowledge of students aged 10–12 attending the ballet school and artistic gymnastics classes. **Conclusions**: The presented nutritional education program may be used as a source of information for specialists for the preparation of educational and repair programs in the group of ballet dancers or artistic gymnasts aged 10–12.

## 1. Introduction

High levels of physical activity generate a greater demand for energy, macronutrients, and some vitamins and minerals. To provide the body with the adequate amount of nutrients, it is necessary to follow nutritional recommendations [1] which consider the proportions of the consumption of groups of products. In the case of children practicing sports, it is also necessary to follow nutritional recommendations related to physical exercise [2]. Adherence to nutritional recommendations in groups of young athletes practicing various sports, including esthetic disciplines, is insufficient and dietary irregularities are often noted. This may lead to inappropriate eating habits which are fixed over time [3,4,5,6,7,8,9]. In addition, ignorance of the main sources of nutrients in food and ignorance of their impact on the functioning of the human body may result in the insufficient intake of proteins, vitamins, minerals, or other nutrients [3]. This problem is particularly important in girls practicing esthetic disciplines, including figure skating, artistic gymnastics, or ballet, for whom it is important to keep a slim figure [9,10]. Nutritional knowledge and the awareness of the need for proper nutrition should be significantly greater in this case, in order to compose nutrient-dense meals including the richest sources of individual nutrients [2,11].

According to the results of the research conducted so far, the nutritional knowledge was found to be insufficient in adolescents practicing sports [3,12]. Consequently, the low level of nutritional knowledge was one of the causes of inappropriate nutritional behavior among young athletes [3,7,8,9,13,14]. The results of the study confirmed [9] that the group of ballet dancers and artistic gymnasts was characterized by a greater incidence of unhealthy eating behavior, which was associated with a rigorous perception of one’s own body and self at the beginning of the professional career, already at the age of 10–12, as well as a higher risk of anorexic readiness. This, in turn, may classify this group as a risk group for developing an eating disorder. Therefore, there is a need for ongoing nutritional education of children and adolescents, to improve their nutritional behavior [13,15,16,17,18], especially in the group of ballet dancers and artistic gymnasts at the beginning of their professional career [9].

Studies involving the nutritional education of ballerinas and gymnasts are sparse and included a group of older girls [19,20,21,22]. However, there is a lack of studies in this field in younger age groups of ballet dancers and artistic gymnasts, although the results of the above studies [9] indicate the need for early nutritional or dietary intervention in this age group. Therefore, the authors of this study attempted to design, implement, and evaluate a nutritional education program in a group of girls aged 10–12 who attended a ballet school and artistic gymnastics classes.

## 2. Materials and Methods

### 2.1. General Information

This article presents the assumptions of the nutritional education program and the results of the assessment of students’ nutritional knowledge just before nutritional education and 3 months after its completion. The research was conducted by a person with a university degree in human nutrition, with many years of experience in conducting this type of research. The consent of the Bioethics Committee at the Food and Nutrition Institute in Warsaw on 7 January 2015, written consent of school heads for the participation of the students in the study, written consent of the legal guardians of the respondents, and the consent of the respondents themselves, were obtained for the study. The research was conducted in accordance with the ethical principles contained in the Declaration of Helsinki. The students and their legal guardians were informed about the purpose of the study and were acquainted with its detailed program [9].

### 2.2. Study Participants

Sixty-five female students aged 10–12 were invited to participate in the study; 60 students at the state ballet school and artistic gymnastics classes professionally practicing ballet and artistic gymnastics (with at least 10 h of training per week) accepted the invitation (92%). Ballet dancers and gymnasts were classified in the same group due to the similar features and the similar nature of ballet and artistic gymnastics. The criteria for exclusion from the study were other age group, the presence of a chronic disease in the students, e.g., diabetes, physical inactivity related to the current injury or disease, and no consent of the legal guardian or the child to participate in the study. All students who gave consent (including parental consent) participated in all stages of the project. No withdrawals were recorded during the study. The characteristics of the study group were published in the article by Leonkiewicz and Wawrzyniak [9].

### 2.3. Evaluation of the Sources of Nutritional Knowledge

We had assessed the sources from which the students obtained their nutritional knowledge prior to starting nutritional education. The question about the sources of nutritional knowledge had 8 possible answers: a doctor, dietitian, teachers/trainers, family, peers, magazines/books, TV/radio, Internet. It was a multiple-choice question. The respondents also self-assessed their nutritional knowledge, stating whether they had a good knowledge of the general principles of nutrition. The students were also asked if they would like to deepen their nutritional knowledge (with yes/no/don’t know answers).

### 2.4. Assessment of Nutritional Knowledge in the Nutritional Education Program

An original questionnaire containing 12 statements divided into 3 sections was used to assess nutritional knowledge. The first section contained 4 statements to verify the knowledge of the principles of the Pyramid of Healthy Nutrition and Physical Activity for Children and Youth (2016) [1]. The second section included 4 statements testing the knowledge concerning the main sources of selected nutrients and their role in the human body [23]. The third section consisted of 4 statements relating to the principles of the nutrition of individuals practicing sports or training [2,24,25]. The pilot study was conducted in 12 girls aged 10–12 who trained artistic gymnastics and ballet to verify the statements. All comments were considered during the preparation of the final version of the questionnaire. The respondents could select the following answers for each statement: T (true), F (false) or DK (I don’t know). Each correct answer was awarded one point. It was possible to obtain the maximum of 12 points. Higher scores indicated higher levels of nutritional knowledge. The average number of points obtained in the nutritional knowledge questionnaire was calculated in the study group, both before and after the nutritional education program. In case of obtaining ≤ 6 points, the level of nutritional knowledge was assessed as lower, for 7–9 points as medium, and for 10–12 points as higher. To assess the effectiveness of the program, an assessment of the level of nutritional knowledge was conducted in a group of ballet dancers and artistic gymnasts just before the start of the nutritional education program and 3 months after its completion.

#### 2.4.1. Nutritional Education Program

**Figure 1:**
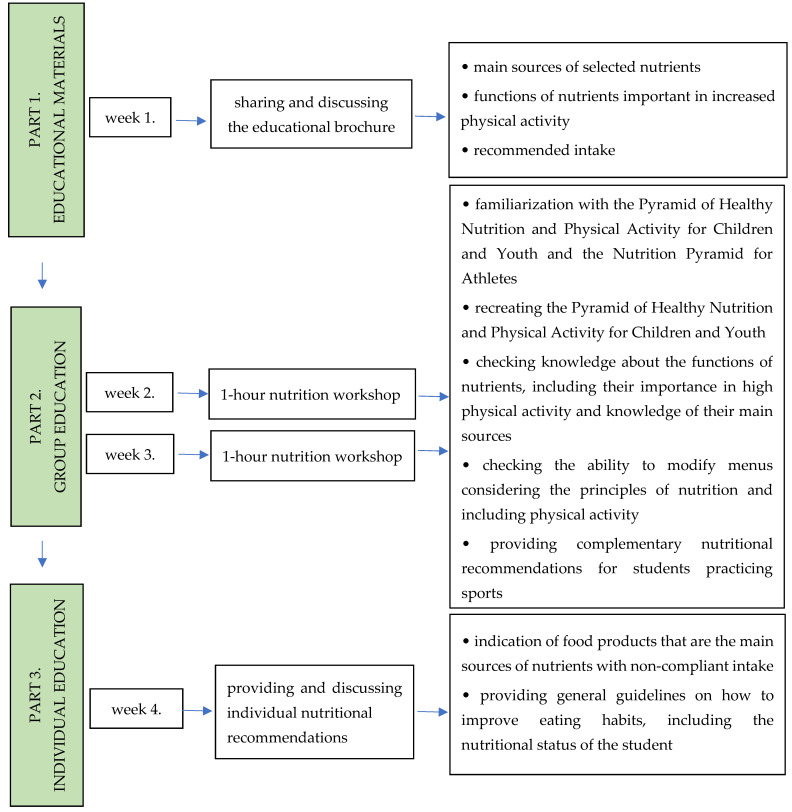
Nutritional education program.

The nutritional education program constituted stage II of the broader project. It was developed on the basis of the results obtained during stage I (initial assessment), taking into account the results of the assessment of the students’ nutritional knowledge obtained in stage I, as well as the results of the assessment of nutritional behavior, nutrient intake and nutritional status of the respondents (Figure S1—Supplementary Material). The information provided to the students during the nutritional education program was related to the statements contained in the nutritional knowledge questionnaire: the principles of proper nutrition included in the Pyramid of Healthy Nutrition and Physical Activity for Children and Youth (2016) [1], the most important sources of nutrients in the diet and their role in the human body [23] and the principles of nutrition of people practicing sports/training [2,24,25]. Group and individual nutritional education consisted of 3 parts: (1) sharing and discussing the educational brochure, (2) participating in group nutrition workshops, and (3) sharing and discussing individual nutritional recommendations (Figure 1). The educational program was carried out over a period of 4 weeks, according to the schedule (Figure 1). Each student took part in 1 h of educational activities per week (group or individual), in each of the 4 weeks.

##### Educational Materials (Brochure)

In the first part of nutritional education (week 1, Figure 1), students received an educational brochure which was then discussed with the group, and important sources of nutrients were indicated. Nutrients presented in the brochure had been selected, considering data showing their importance in increased physical activity, including children and adolescents practicing esthetic disciplines, or due to their insufficient intake (Figure S2—Educational materials, brochure). The brochure presented the main sources of protein, omega-3 acids, calcium, iron, magnesium, potassium, zinc, iodine, folate, vitamin C, vitamin D, vitamin E with the content of the above-mentioned nutrients per 100 g of selected products [26,27]. In addition, the daily requirement of the above-mentioned nutrients and water in relation to the reference ranges (RDA or AI) for girls aged 10–12 was also presented [1]. In relation to protein, an intake recommendation for high-activity students was provided [24,25]. Moreover, information on the impact of each nutrient on the human body was presented, with an emphasis on the importance during physical exertion [1,23]. The sources of the presented data included leading Polish publications/books, which constitute the substantive base for students and scientists in the field of human nutrition [1,2,23,24,25,26,27].

##### Group Education (Nutritional Workshops)

The second part of nutritional education involved participating in nutritional workshops (two 1 h group meetings) conducted independently with different age groups (10, 11, and 12 years). The aim of the workshop was to provide knowledge about the general principles of proper nutrition, as well as the principles of nutrition for students with high levels of physical activity. The workshop consisted of 3 practical tasks.

The aim of the first meeting (week 2, Figure 1) was to familiarize students with the Pyramid of Healthy Nutrition and Physical Activity for Children and Youth developed by the Food and Nutrition Institute in Warsaw [1], which was discussed in detail with the presentation of 10 principles of proper nutrition [1]. In 2019, the Pyramid was supplemented with elements of a healthy lifestyle [28]. However, the arrangement of food products in the Pyramid and the accompanying principles of proper nutrition remained unchanged. In addition, the Nutrition Pyramid for Athletes was shown to the students [25]. The Nutrition Pyramid for Athletes distinguishes the groups of products that should be consumed in additional amounts as the hours of training increase. It was emphasized that taking account of the above recommendations (contained in the pyramids) creates optimal conditions for maintaining health, increasing physical efficiency and, thus, achieving sporting success. Then, the students were divided into pairs and asked to perform two tasks. The first task was to recreate the food pyramid from memory, using a mix of photos of products from different groups. The arranged pyramids were then discussed with the group. The second task was to match the names of selected nutrients to their functions in the human body, considering the needs of the body during increased physical activity. The results of the second task were discussed with the participants.

The second meeting, which was held a week later (week 3, Figure 1), began with a revision of information from the previous meeting regarding the function of nutrients. Subsequently, the girls listed those food products which, in their opinion, were the richest sources of nutrients. The emerging doubts were explained, and the errors were corrected in the discussion. At the end of this activity, the girls chose 2 nutrients they found the most interesting due to their functions in the human body. Then, they used the photos used in the first task (a mix of product photos from different groups) to indicate the products they thought were their main sources. Next, they exchanged their insights in the discussions between the groups. The third practical task carried out during the workshop was the assessment and modification of a 3-day menu of a person with high physical activity levels. The menu was verified, taking into account the principles presented in the Pyramid of Healthy Nutrition and Physical Activity for Children and Youth. The students’ task was to modify the sample menu so that each item was correct, e.g., adding missing products, removing products that were not recommended, and the most complex task involving replacing one product with another. In addition, 2 out of 3 days were to include training after breakfast/snack, which also had to be considered in the modification of the menu. This part of the workshop was supplemented with information on the importance of consuming the recommended number of meals during the day, eating breakfast, composing meals, and the principles of composing meals before and after training.

##### Individual Education (Individual Dietary Recommendations)

Based on stage I (initial assessment), individual nutritional recommendations were developed for each student (week 4, Figure 1), taking account of the results related to nutritional behavior, nutrient intake and nutritional status (Figure S1—Supplementary Material). Each student was provided with guidelines concerning changes in nutrient intake and the products that included them to balance nutritional inadequacy. The authors of the program also proposed changes in the consumption of product groups in accordance with the recommendations of the Pyramid of Healthy Nutrition and Physical Activity for Children and Youth. Individual recommendations also referred to the individual nutritional status, i.e., the body fat content and BMI of the respondents [9]. To facilitate understanding, the process of preparing individual dietary recommendations and how to interpret them were discussed. Subsequently, the person conducting the study explained the individual doubts and answered additional questions.

### 2.5. Statistical Analysis

Statistical analysis was performed using Statistica 13.0 (StatSoft Polska) statistical software. To verify compliance with the normal distribution, the Shapiro–Wilk test was used. Categorical variables, such as nutritional knowledge before and after the education program, were compared with the Cochran’s test, also for the following categories—lower, medium, higher. The Wald test refers to the state of the knowledge before education, for which the odds ratio was set at OR = 1. For quantitative variables, the mean and standard deviation were calculated. The sums of points in the assessment of nutritional knowledge before and after education were compared with the Wilcoxon test (for means). The size of the r-Wilcoxon effect was calculated for the groups (r < 0.3 small effect, 0.3 < r < 0.5 medium effect, r ≥ 0.5 large effect). The level of statistical significance for the evaluation of the results was set at α = 0.05 (two-tailed level), and a statistical trend was determined for *p* ≤ 0.1.

## 3. Results

**Figure 2:**
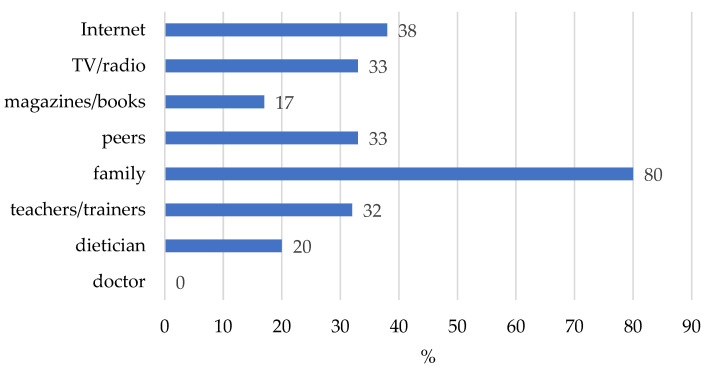
Sources of nutritional knowledge of the students at a ballet school and artistic gymnastics classes before starting the nutritional education program.

As indicated by 80% of respondents, in the group of girls aged 10–12 practicing ballet and artistic gymnastics, the main source of nutritional knowledge before starting the nutritional education program was the family (Figure 2). The mass media, such as the Internet, radio/television, also constituted important sources (38% and 33%, respectively). Moreover, 1/3 of the girls obtained nutritional knowledge from the closest school environment—from the teachers and trainers, as well as from their peers (32–33%). None of the respondents indicated a doctor as the person from whom they obtained nutritional knowledge. Only 20% indicated a dietitian and 17% indicated magazines/books. Over half of the respondents (57%) stated that they had a good knowledge of the general principles of proper nutrition, and 72% showed a willingness to broaden their nutritional knowledge.

The highest percentage of correct answers to individual statements was found in the third section of the statements relating to the principles of nutrition of people practicing sports (Table 1). The number of correct answers in this part ranged from 73–90% before the education to 77–95% after the education. A lower frequency of correct answers was noted in the first section of the statements relating to the principles of the Pyramid of Healthy Nutrition and Activity for Children and Youth, and the lowest in the second section, i.e., assessing the knowledge about the sources of nutrients and their role in the human body. In the second section, before the education, half of the girls had not known that (fatty) fish was the main source of vitamin D in the diet and that nuts were the source of fats with a beneficial effect for health. As many as 75% of the respondents incorrectly indicated vegetables and fruit as the main sources of iron in the diet.

Comparing the respondents’ answers to the same statements before and after the education, it may be concluded that a statistically significant improvement was observed in case of statements related to the source of vitamin D (*p* = 0.008), the source of fats that are beneficial for health (*p* = 0.008), and the source of iron (*p* = 0.002). Significant changes in the number of correct answers were also found in case of knowledge of the principles relating to the frequency of fruit (*p* = 0.025) and milk and dairy product (*p* = 0.046) consumption. The share of correct answers to all questions increased from 55 to 61% in the first section, from 47 to 58% in the second section, and from 84 to 88% in the third section. In general, the greatest increase (11%) in the percentage of correct answers after nutritional education was observed in the second section. Moreover, the share of individuals with a lower level of nutritional knowledge decreased (*p* = 0.025), and the share of those with a higher level of knowledge increased (*p* = 0.005).

The total average number of points obtained for all statements of the nutritional knowledge test after nutritional education was significantly higher than the result before the education (*p* < 0.0001; Wilcoxon effect size = 0.61) (Table 2). In addition, taking account of the average number of points obtained for each section, the highest increase was observed in the second section, assessing the knowledge of nutrient sources and their role in the human body (*p* < 0.001; Wilcoxon effect size = 0.50), where the knowledge score before starting the education was the lowest.

## 4. Discussion

Our study showed that our nutritional education program influenced the nutritional knowledge of ballet and artistic gymnastics students aged 10–12 years, indicating an area of knowledge that requires special attention in educational and intervention programs for younger ballet and artistic gymnastics students. Therefore, in addition to knowledge about nutritional recommendations which are crucial under conditions of increased physical activity [3,14,29], nutritional education conducted in this group should also provide knowledge about the richest sources of nutrients that are especially important in physically active individuals [30,31].

### 4.1. Sources of Nutritional Knowledge

The results of research conducted in older children and adolescents practicing sports, including gymnastics, indicated that the family was the main source of nutritional knowledge [12]. Similar results were obtained in the present study. However, the percentage of the respondents indicating the family as a source of nutritional knowledge was higher in the group of younger girls, which may be related to the fact that the family authority was more important in this group. The present study also revealed that younger, physically active girls indicated their trainer as a person communicating nutritional knowledge less often than it was observed in older athletes [10]. Therefore, the above results confirm the need for the nutritional education of parents and trainers/teachers [4,32], who provide young athletes with knowledge about the principles of nutrition. The attitude of the respondents is also an important aspect in education. The study demonstrated that 72% of the respondents showed a willingness to broaden their nutritional knowledge. The need was significantly lower in older athletes [33], which confirms the necessity to conduct nutritional education in the youngest age group as soon as possible.

### 4.2. Forms of Nutritional Education

Nutritional education conducted in a group of children should take various forms, such as workshops and lectures, to make it attractive and effective [21,34,35,36,37]. The differentiation of colors in the transfer of knowledge may help to focus the attention on important information [38], hence the use of a color brochure in the implemented nutritional education program. Group nutrition workshops, during which the girls might demonstrate their acquired knowledge in practice and discuss their doubts, is another important stage of education. Groupwork can stimulate motivation to acquire knowledge [39] and learning through experience increases the effectiveness of this process [37]. Individual recommendations are an important summary element, increasing awareness of the importance of proper nutrition in young sportswomen. Such recommendations may also be used by parents and trainers to verify the nutrition of an athlete and to individually correct nutritional mistakes. The study in the group of adults showed that individual education might be more effective in improving anthropometric parameters compared to group education [40]. As regards educational activities conducted among children and adolescents practicing sports [16,20,21,34,41], our research covered a shorter period of nutritional education but, importantly, a variety of forms were offered. However, as shown in the study by Doyle-Lukas and Davy [42], shorter-term education also contributed to the improvement of factors such as the level of the nutritional knowledge of the respondents.

### 4.3. Reference Materials and Important Topics of Nutritional Education in the Group of Ballet and Artistic Gymnastics Students

The knowledge of the principles of the Pyramid of Healthy Nutrition and Physical Activity of Children and Youth [1] was the primary source of nutritional knowledge in the implemented educational program. It constitutes the basis for the correct composition of meals both for children practicing sports and those not practicing sports. The authors of Polish and foreign studies often use food pyramids as reference materials in such programs [17,37,43,44].

The transfer of knowledge concerning the sources and role of nutrients is the field that requires special consideration in the preparation of educational and intervention programs dedicated to students of younger classes of ballet schools and artistic gymnastics.

Particularly important principles of the nutrition of children practicing sports include the adequate consumption of milk and dairy products, i.e., 3–4 servings a day [1,25]. Although the knowledge of this principle increased significantly after the education, 42% of girls did not acquire proper knowledge about it. Considering the inconsistent consumption of dairy products in the group of training children [45] and the insufficient consumption of calcium (with dairy products being its main source), detailed education in this area is necessary [9]. Another important recommendation is related to the consumption of fruits and vegetables. The knowledge that fruit should be consumed every day [1,25] was not acquired by 27% of the girls. It may be due to the fact that girls training esthetic disciplines, which require the maintenance of a specific body weight, are convinced of the need to avoid products that are a source of carbohydrates, including simple sugars [9,20]. Debnath et al. [14] observed that only 55% of their study group of hockey and football players aged 14–19 were aware that the daily consumption of fruit and vegetables was important in terms of providing vitamins and minerals. Another study showed that 90% of football players aged 17–19 believed that fruit and vegetables were a source of “empty calories” [46].

The fewest correct answers were provided by the group of young ballerinas and artistic gymnasts in the section concerning the knowledge of the basic sources of nutrients and their role in the human body. Such a trend was also reflected in the results obtained by older adolescents practicing various disciplines [47]. This confirms the necessity to conduct nutritional education in younger girls as early as possible to eliminate nutritional mistakes because of insufficient knowledge in this area [3,13,14]. A study by Kostanjevec et al. [36] was conducted in children of the same age but ones who were not training. The authors also reported a problem with determining the nutritional value of products, which might be related to the lack of knowledge about individual nutrients. The lack of awareness that fatty fish is the main source of vitamin D combined with commonly insufficient intake of this ingredient in the diet [48] may be a serious problem, considering the pleiotropic role of vitamin D in the body [49,50]. Low supply of vitamin D is also not conducive to training, including indoor training, typical in the group of ballet dancers and gymnasts, which additionally limits the ability to synthesize vitamin D in the body under the influence of sunlight [50]. The incorrect indication of fruits and vegetables as the main sources of iron by 75% of the girls in the present study might be related to the common belief that spinach is rich in iron [12]. Cupisti et al. [12] conducted a study in female athletes (including gymnasts) aged 14–18 and reported a slightly higher percentage of correct answers concerning the main sources of iron. However, over half of the girls had a knowledge deficit in that area, indicating spinach or bread as sources of iron [12]. The knowledge of foods rich in different types of dietary fats was also problematic. Bird and Rushton [10] demonstrated that approximately 70% of older athletes (aged 13–18) incorrectly stated that avocado had a low fat content. Figurska-Ciura et al. [46] observed that over half of young football players indicated butter as the source of essential fatty acids. In regard to our study, about half of our respondents failed to indicate nuts as a source of fats that was beneficial for the health. Girls practicing esthetic disciplines requiring the maintenance of a certain body weight may view fats as something that should be avoided. This was confirmed by the results of a previous study showing the opinion of the respondents that limiting the consumption of fats and carbohydrates was associated with feeling dissatisfied with oneself and the belief that appearance was extremely important in achieving life success [9].

As regards our study, the most correct answers were recorded for the statements concerning the knowledge of the principles of nutrition for individuals practicing sports. Conversely, other authors noted lower levels of knowledge regarding the principles related to hydration [14,47,51]. A large proportion of young people practicing sports were also unaware of the recommended break between the last meal and training, or believed that diet was less important outside the training period [14,47].

### 4.4. Results of Implementing Nutritional Programs

The proposed nutritional education program had a significant impact on the level of nutritional knowledge of students aged 10–12 attending ballet school and artistic gymnastics classes, identifying an area of knowledge that requires particular attention. In the programs conducted so far, Nascimento et al. [17] achieved an improvement in nutritional knowledge after implementing a nutritional intervention among teenage athletes, but only in terms of knowledge about the principles of the food pyramid, without significant changes in the area of basic aspects of nutrition and principles of nutrition for athletes. In turn, Aguilo et al. [18] did not observe any improvement in nutritional knowledge in a group of teenage artistic gymnasts from Iceland who participated in an 8-month nutritional education program. Similar results were obtained by Visiedo et al. [22], who did not observe any improvement in nutritional knowledge in the group of artistic gymnasts under 18 years of age, which may be due to the high level of nutritional knowledge in this group (over 75%), found before the start of the educational program.

### 4.5. Strengths and Limitations of the Study

To our knowledge, no nutritional education program dedicated to the younger ballet dancers and artistic gymnasts with an assessment of its effectiveness, including individual components, has been described in the literature. The research carried out so far has included older or heterogeneous age groups of respondents [8,18,19,20,21,34]. Moreover, previous studies conducted in young athletes mainly focused on the general assessment of whether nutritional knowledge improved after education [34,41]. The present study additionally indicates the area of nutritional education which should be particularly tackled in the group of 10–12-year-old ballerinas and gymnasts. This may suggest a further direction for the development of educational and repair programs in this group of children. The nutritional education program was conducted by a qualified dietitian, based on reference sources of knowledge.

The study duration may constitute a limitation of the study. Increasing the period of nutritional education in the group of young ballerinas and artistic gymnasts aged 10–12 could improve its effectiveness. Nutrition knowledge was assessed using 12 selected statements, which could lead to underestimating or overestimating the effects of the program in assessing nutrition knowledge.

The results of this study may complement the studies of other authors conducted in other age groups and may be helpful in developing nutritional education for athletes at the beginning of their careers.

## 5. Conclusions

The proposed nutritional education program had a significant impact on the level of nutritional knowledge of ballet and artistic gymnastics students aged 10–12. Knowledge about the sources and role of nutrients is an area that requires special consideration in the preparation of educational and intervention programs tailored for students of younger classes of ballet schools and artistic gymnastics. In addition to educating students, nutritional education should also include the families of ballet dancers and gymnasts aged 10–12 and their teachers and coaches, as the closest environment from which the girls acquire their nutrition-related knowledge.

## Figures and Tables

**Table 1:** Nutrition-related knowledge of the students at a ballet school and artistic gymnastics classes before and after the nutritional education program.

Statements in Sections (S)	Correct Answer	Education		*p* ^(1)^	OR (95% Cl) ^(2)^
		% of Students with the Correct Answer (n = 60)			
		Before	After		
Knowledge of the principles of the Pyramid of Healthy Nutrition and Physical Activity for Children and Youth					
S1: Cereal products (bread, pasta, rice, groats) should be the main sources of energy in each meal	Yes	72	73	0.317	1.087 (0.484–2.444)
S2: Fruit should be eaten every other day	No	65	73	0.025 **	1.481 (0.673–3.257)
S3: Milk and dairy products (yogurt, kefir, cheese) should be consumed several times a day	Yes	52	58	0.046 **	1.309 (0.632–2.713)
S4: Vegetable fats should be present in the diet in lower amounts than animal fats	No	33	40	0.103	1.333 (0.628–2.830)
Knowledge of the main sources of selected nutrients and their role in the human body					
S5: Vegetables and fruits are the main sources of iron in the diet	No	25	42	0.002 **	2.143 ** (0.976–4.703)
S6: Nuts are a source of fats that are beneficial for health	Yes	52	63	0.008 **	1.616 ** (0.773–3.377)
S7: (Fatty) Fish is the main source of vitamin D in the diet	Yes	48	60	0.008 **	1.603 (0.772–3.330)
S8: 100 g of meat provides less protein than 100 g of potatoes	No	63	67	0.158	1.158 (0.542–2.473)
Knowledge of the principles of nutrition for students practicing sports/training					
S9: The main meal (e.g., lunch) should be eaten just before exercise	No	87	88	0.317	1.160 (0.389–3.483)
S10: Fat-and-carbohydrate snacks (e.g., a chocolate bar) are good snacks during training (dance classes)	No	73	77	0.157	1.195 (0.518–2.758)
S11: After intensive training/exercise, first of all, water losses should be replaced	Yes	90	95	0.083 *	2.111 (0.496–8.993)
S12: Playing sports (ballet) increases the need for nutrients	Yes	87	90	0.157	1.385 (0.444–4.314)
Nutrition-related knowledge (% of students)					
lower (≤6 points)		22	13	0.025 **	
medium (7–9 points)		67	60	0.285	
higher (10–12 points)		12	25	0.005 **	

^(1)^ the Q Cochran’s test; ^(2)^ the Wald test (OR value); * p ≤ 0.1—statistical tendency; ** *p* ≤ 0.05—statistical significance.

**Table 2:** Assessment of nutrition-related knowledge of the students at a ballet school and artistic gymnastics classes before and after the nutritional education program.

Assessment of Nutrition-Related Knowledge	Education Total Points (n = 60)		*p* ^(1)^
	Before	After	
S: S1–S4 (max 4 points)	2.2 ± 1.1	2.5 ± 1.0	0.005 *
Wilcoxon effect size = 0.36			
S: S5–S8 (max 4 points)	1.9 ± 1.1	2.3 ± 1.1	<0.001 *
Wilcoxon effect size = 0.50			
S: S9–S12 (max 4 points)	3.4 ± 0.8	3.5 ± 0.7	0.027 *
Wilcoxon effect size = 0.28			
S: S1–S12 (max 12 points)	7.5 ± 1.8	8.3 ± 1.8	<0.0001 *
Wilcoxon effect size = 0.61			

S—Statements from Table 1; ^(1)^ Wilcoxon test; * *p* ≤ 0.05—statistical significance.

## Data Availability

The dataset used and/or analyzed during this study is available from the corresponding author upon reasonable request. The data are not publicly available due to ethical restrictions and participant confidentiality.

## Supplementary Materials

The following supporting information can be downloaded at https://www.mdpi.com/article/10.3390/nu17091468/s1; Figure S1: Scheme of the project; Figure S2: Educational materials (brochure). References [1,2,23,24,26,27] are cited in Supplementary Materials.
