# Supplementary material for: Evaluation of the Nutritional Education Program in Increasing Nutrition-Related Knowledge in a Group of Girls Aged 10–12 Years from Ballet School and Artistic Gymnastics Classes
Source: Nutrients. 2025 Apr 26;17(9):1468. doi: 10.3390/nu17091468 (PMC12073703; doi:10.3390/nu17091468)
Supplement: Supplementary file 1 [file nutrients-17-01468-s001.zip › Figure S1.pdf]

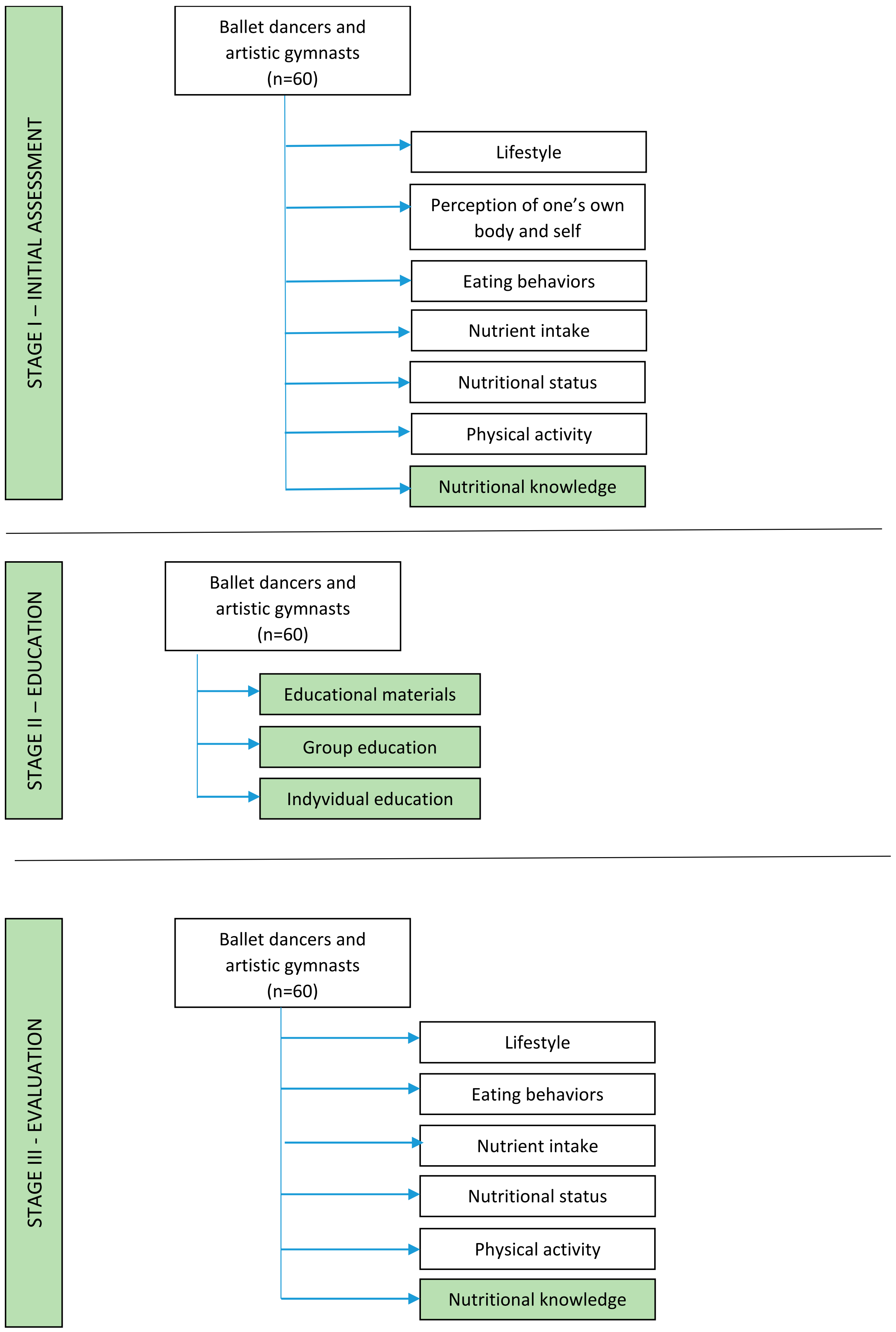


**Figure S1.** Scheme of the project for assessing the impact of nutritional education on changes in the diet, nutritional status and nutrition-related knowledge of ballet school students and artistic gymnastics classes. The stages highlighted in colour refer to the content of the results presented in this study.
