# Supplementary material for: Evaluation of the Nutritional Education Program in Increasing Nutrition-Related Knowledge in a Group of Girls Aged 10–12 Years from Ballet School and Artistic Gymnastics Classes
Source: Nutrients. 2025 Apr 26;17(9):1468. doi: 10.3390/nu17091468 (PMC12073703; doi:10.3390/nu17091468)
Supplement: Supplementary file 1 [file nutrients-17-01468-s001.zip › Figure S2.pdf]

NUTRITION KNOWLEDGE IMPORTANT FOR A YOUNG BALLERINA!

NUTRIENTS	Nutritional requirements per day – girls 10-12 years
**PROTEIN** - **builds** the body, including **muscles**, – is responsible for the **growth and development**.	**1.1 – 2.0 g/kg body weight**

The amount of **PROTEIN** in 100 g of the product

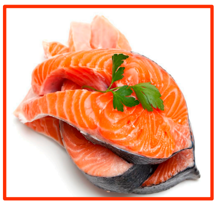

20 g (raw)

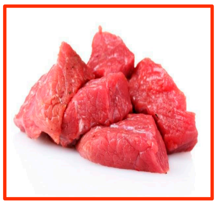

16 – 21 g (raw)

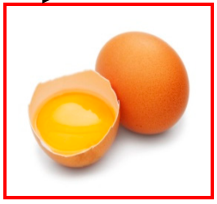

13 g (raw)

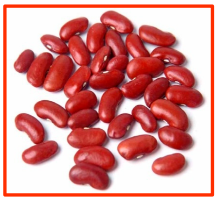

21 g (dry)

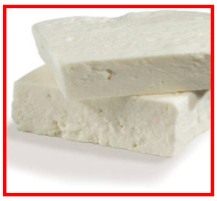

18 - 20 g

OMEGA-3 ACIDS - participate in the proper functioning of the **brain**,
- regulate the function of the **retina**, - are a component of **cell membranes**.

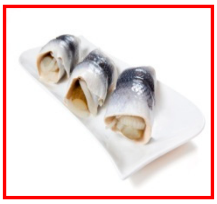

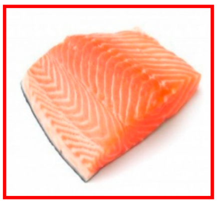

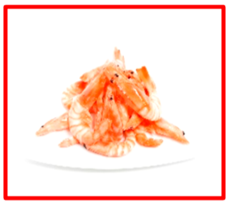

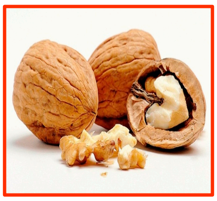

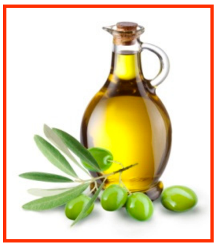

**CALCIUM** - builds strong **bones and teeth**, - is involved in the proper **functioning** of the **muscles** and the **nervous system.**	**1300 mg**

The amount of **CALCIUM** in 100 g of the product

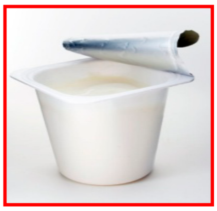

170 mg

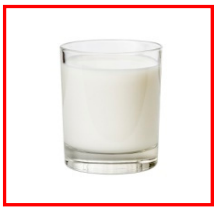

120 mg

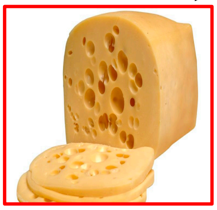

530 - 970 mg (various cheeses)

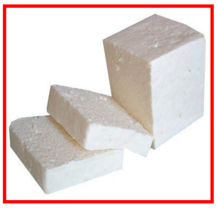

90 - 100 mg

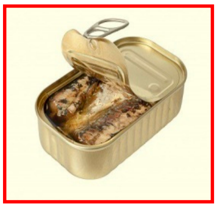

330 mg (sardines)

**IRON** - as a component of hemoglobin (blood pigment), - it **reduces fatigue** during exercise, - **increases immunity**.	**10 mg**

The amount of **IRON** in 100 g of the product

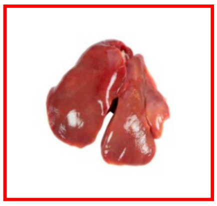

8 – 19 mg (various livers)

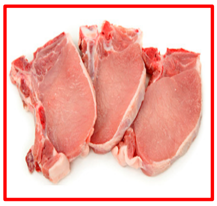

1 - 3 mg (raw)

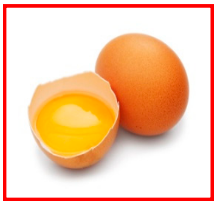

2 mg (raw)

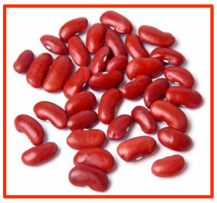

7 mg (dry)

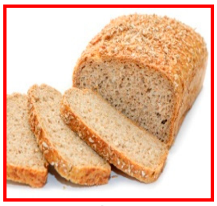

1 – 3 mg

MAGNESIUM - necessary for proper **muscle contraction**,
- participates in signal transmission in the **nervous system**.

240 mg

The amount of **MAGNESIUM** in 100 g of the product

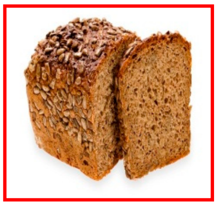

40 – 70 mg
(wholemeal)

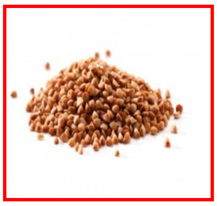

218 mg
(buckwheat groats)

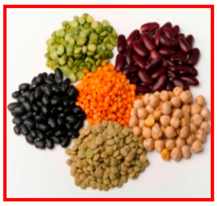

120 – 220 mg
(dry legumes)

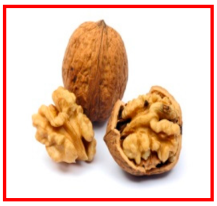

100 – 180 mg
(various nuts)

POTASSIUM - participates in regulating **blood pressure**,
- is involved in **muscle contraction** and **nerve conduction**.

2400 mg

The amount of **POTASSIUM** in 100 g of the product

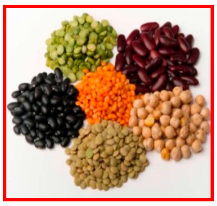

850 – 2000 mg
(dry legumes)

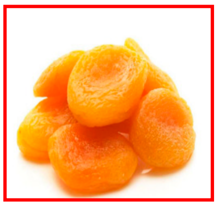

1700 mg

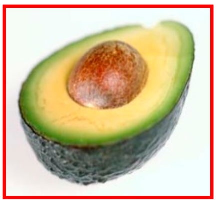

600 mg

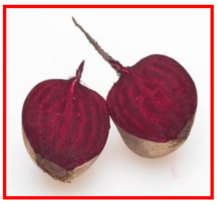

350 mg

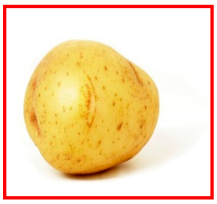

420-490 mg

ZINC - necessary for the functioning of many **hormones**,
- **reduces** the risk of **infection** in people with high physical activity.

8 mg

The amount of **ZINC** in 100 g of the product

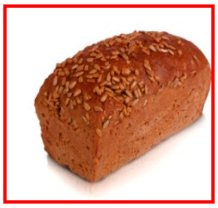

1 – 3 mg

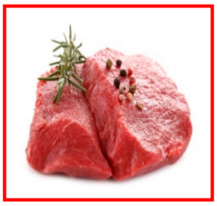

1 – 4 mg
(raw)

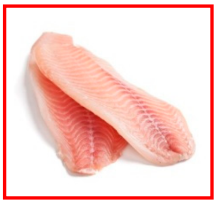

0,5 – 2 mg
(raw)

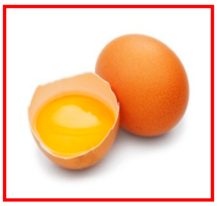

2 mg
(raw)

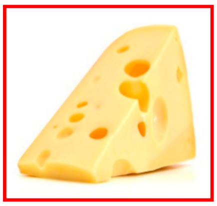

2 – 4 mg
(various cheeses)

IODINE - necessary for the synthesis of **thyroid hormones**, which affect the development of **muscles (heart)**, cell maturation, **metabolism** and maintaining body **temperature**.

120 µg

The amount of **IODINE** in 100 g of the product

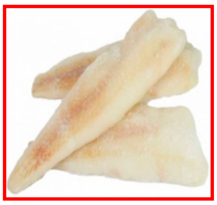

100 µg
(raw pollock)

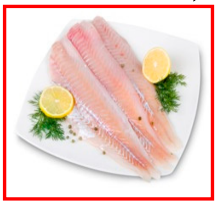

110 µg
(raw cod)

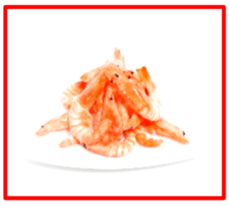

40 µg
(raw)

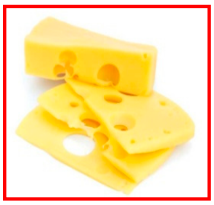

30 – 40 µg

FOLATE - necessary for DNA synthesis, cell division,
- are involved in the functioning of the nervous and hematopoietic systems

300 µg

The amount of **FOLATE** in 100 g of the product

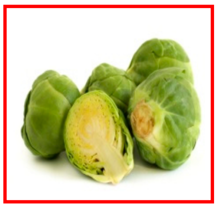
130 µg

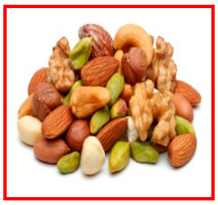
60 – 110 µg
(various seeds)

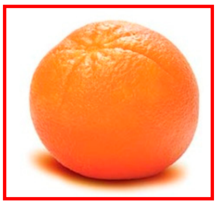
30 µg

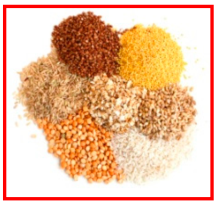
20 – 50 µg
(dry groats)

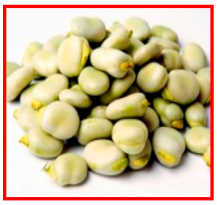
150 µg
(broad beans)

VITAMIN C - protects the body **against free radicals** that are formed during exercise, - supports **wound healing**.

50 mg

The amount of **VITAMIN C** in 100 g of the product

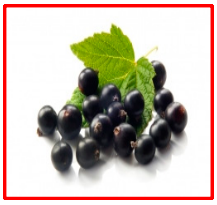
180 mg

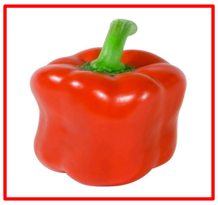
140 mg

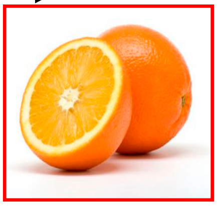
50 mg

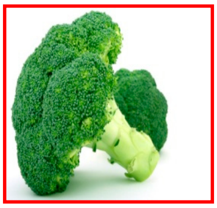
80 mg

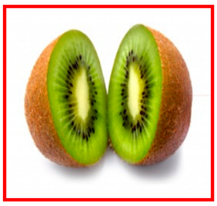
60 mg

VITAMIN D - participates in **building bone tissue,**
- helps in the proper **functioning of muscles**.

15 µg

The amount of **VITAMIN D** in 100 g of the product

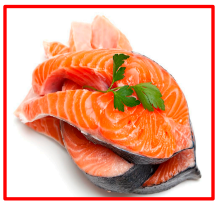
13 µg
(raw)

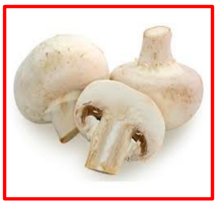
2 µg

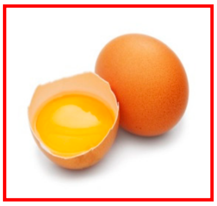
2 µg
(raw)

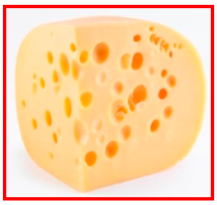
0.2 – 0.7 µg
(various cheeses)

VITAMIN E - **protects against free radicals** during physical exertion,
- affects the **proper performance of the muscles**.

8 mg

The amount of **VITAMIN E** in 100 g of the product

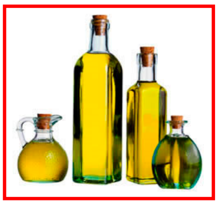
12 – 50 mg
(various oils)

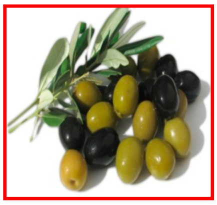
2 mg

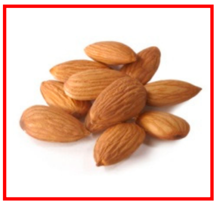
24 mg

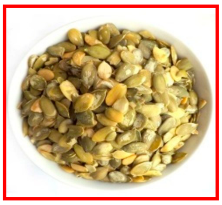
26 mg
(pumpkin seeds)

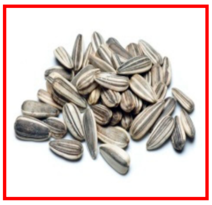
28 mg
(sunflower seeds)

WATER - participates in **digestion, metabolism**,
- enables **joint mobility**, regulates **body temperature**.

2 l per day + 0.4 – 0.8 l/ 1 hour of exercise

COMMENT:

MAGNESIUM AND CALCIUM - highly mineralized water is also a good source.

IRON - it is absorbed much better from meat products (heme iron) than from plant products (non-heme iron).

IODINE - iodized salt is also a good source.

VITAMIN C - during thermal processing (cooking, frying, baking, etc.) there is a large loss of this vitamin, so it is recommended to eat fruit and vegetables raw (if it is possible).

VITAMIN D - in April - October is also produced in human skin.

WATER - in addition to liquids, products such as soups, yoghurts, fruit and vegetables are also a good source of water.

Prepared on the basis:

- *Jarosz, M. (ed.). Nutrition recommendations for the Polish population. Institute of Food and Nutrition, Warsaw, Poland, 2017.*
- *Kunachowicz, H.; Nadolna, I.; Przygoda, B.; Iwanow, K. Tables of food composition and nutritional value. PZWL Publishing, 1st ed., Warsaw, Poland 2005.*
- *Kunachowicz, H.; Nadolna, I.; Przygoda, B.; Iwanow, K. Tables of food composition and nutritional value. PZWL Publishing, 2nd ed., Warsaw, Poland 2017.*
- *Gawęcki, J. (ed.) Human Nutrition. Basics of the science of nutrition. PWN Publishing, Warsaw, Poland 2012.*
- *Smith, J.W.; Holmes, M.E.; McAllister, M.J. Nutritional considerations for performance in young athletes. J. Sports Med. (Hindawi Publ Corp) 2015, 2015:734649.*
- *Bonci, L. Sports nutrition for young athletes. Pediatr. Ann. 2010, 39, 300-306.*
